# The effect of gamma irradiation on astaxanthin synthetase encoding gene in two mutant strains of *Phaffia rhodozyma*

**Published:** 2013-09

**Authors:** Naeimeh Najafi, Ramin Hosseini, Ali-Reza Ahmadi

**Affiliations:** 1 Women Research Centre, BioMedical Sciences Department, Alzahra University, Tehran, Iran; 2 Agricultural Biotechnology Department, Faculty of Engineering, Imam Khomeini International University, Qazvin, Iran

**Keywords:** Astaxanthin, *Phaffia rhodozyma*, yeast, Astaxanthin synthetase, cytochrome P450

## Abstract

**Background and Objectives:**

Astaxanthin, an orange-red carotenoid pigment, acts as a protective agent against oxidative damage to cells in vivo. The astaxanthin synthetase gene (*crtS*) size consists of 3995 bp. This gene has been suggested to catalyse β-carotene to astaxanthin in *Phaffia rhodozyma*. The aim of this research was to find any possible changes in this gene in two mutant strains, Gam1 and Gam2 (with high astaxanthin pigment production), previously created by gamma irradiation.

**Materials and Methods:**

The astaxanthin synthetase gene sequence of *Phaffia rhodozyma* in the NCBI Gene bank was used to design primer. In Gam1, this gene was amplified using primers Asta F_1_, Asta R_2_, Asta F_3_, Asta R_4_. In Gam2, primers asta F_1_, asta R_4_ were used to amplify the gene. The amplified fragments were 8 sequenced using primers Asta F_1_, Asta R_1_, Asta F_2_, Asta R_2_, Asta F_3_, Asta R_3_ and Asta F_4_, Asta R_4_. Astaxanthin synthetase gene from two mutant strains, Gam1 and Gam2 were amplified using PCR. The amplified products were sequenced and aligned using the ClustalW software.

**Conclusion:**

The comparison of this gene showed 98% and 99% similarities between the reference sequence and Gam1 and Gam2 mutant strains, respectively, whereas the comparison of this gene in Gam1 and Gam2 mutant strains showed 97% similarity. However, the deduced proteins showed 78% and 83% between the reference protein obtained from the wild type and Gam1 and Gam2, respectively. This similarity was 75% between the mutant strains.

## INTRODUCTION

Carotenoids play an important role as colorants, feed supplements, and nutraceuticals in the food, medical, and cosmetic industries. More than 600 different chemical structures have been described (1). Carotenoids in general are able to eliminate free radical-containing agents produced in the cells (2). They are terpenoids with the isopentenyl-pyrophosphate (IPP) molecules as the basic unit (3). *Xanthophylomyces dendrorhous* (*Phaffia rhodozyma*) and *Haematococcus pluvialis* are currently known as the main microorganisms useful for astaxanthin production at the industrial scale (4–6). Additionally, some marine bacteria, including *Paracoccus* sp. have been shown to be able to biosynthesize astaxanthin and are being checked as an alternative for astaxanthin production (7, 8).

Astaxanthin is an abundant carotenoid pigment responsible for the color of the flesh of many marine species by the ingestion of microorganisms that synthesize the pigment. Although more than 600 different cartenoids have been discovered from the carotenogenic microorganisms, only ß-carotene, lycopene and astaxanthin are commercially produced by microbial fermentation (9). The antioxidant activity of astaxanthin is about 100-500 times that of vitamin E (10). Its antioxidant properties have increased the industrial interest on the uses of astaxanthin in the aquaculture, chemical, pharmaceutical, and alimentary industries (11).

The proposed biosynthetic route of astaxanthin in *Phaffia rhodozyma* is isopentenyl- pyrophosphate (IPP) → geranyleranyl pyrophosphate (GGPP) → phytoene → lycopene → β-carotene → astaxanthin. Recently, it has been published that the conversion of β-carotene into astaxanthin requires only one enzyme, astaxanthin synthetase or CrtS, encoded by *crtS* gene. This enzyme belongs to the cytochrome P450 protein family (12).

Two enzymatic activities convert β-carotene into astaxanthin through several biosynthetic intermediates: a ketolase which incorporates two 4-keto groups in the molecule of β-carotene, and a hydroxylase which introduces two 3-hydroxy groups. In *X. dendrorhous*, both activities could be together as a single enzyme (astaxanthin synthetase; CrtS) encoded by the *crtS* gene, which sequentially catalyzes the 4-ketolation of β-carotene followed by the 3-hydroxylation (13).

Mutagenesis by gamma rays studies in order to obtain microorganisms with the higher capabilities has been the subject of various studies *e.g*. in *Hansenula anomala* and *Rhodotorula rubra*, *Phaffia rhodozyma* and *Aspergills niger* (14–16). In the previous paper using gamma rays on *Phaffia rhodozyma* we obtained 11 mutant strains, two of which were astaxanthin overproducers (17). RAPD markers showed a relatively high polymorphism in the mutant strains (18). Here, we explain changes occurred in the key enzyme in astaxanthin biosynthetic pathway, astaxanthin synthase encoding gene (*ctrS*), in two astaxanthin overproducing mutant strains of *Phaffia rhodozyma*.

## MATERIALS AND METHODS

This study was carried out from 2008-2010 at the Department of BioMedical Sciences, Women Research Center, Alzahra University. Gamma irradiation has been done at the Radiancy Applications Research Institute, Tehran, Iran. Gamma rays were used to induce mutagenesis with 1, 2, 3, 3.5, 4, 4.5, 5, 5.5, 6 and 7 kGy (18). Several mutant strains were obtained at all doses and two astaxanthin overproducing mutant strains, designated Gam1 and Gam2 were chosen and used for this study. In this paper we are reporting the findings on the structural changed occurred in astaxanthin synthase gene in these mutant strains, using PCR, sequencing and aligning sequences with the reference sequence derived from the wild type.

### Yeast culture, Genomic DNA extraction

The strains were grown in YM broth medium (10 g/L glucose, 5 g/L bactopeptone, 3 g/L yeast extract, 3 g/L malt extract; Difco) at 20 °C in a shaker incubator (140 rpm) for 24h. Total DNA was isolated by a modification of the method of Chung (19). The quantity of DNA was measured by spectrophotometry at 260 and 280 nm and by electrophoresis on a 1% (w/v) agarose gel.

### Primer design and astaxanthin synthetase gene amplification

Astaxanthin synthetase gene in the reference and the mutant strain Gam1 was amplified using two pairs of primers. A pair of primers included Asta F1 as the forward primer and Asta R2 was used as the reverse primer. Other pair of primers included Asta F3 as the forward and Asta R4 as the reverse primer. Also astaxanthin synthetase gene was amplified in mutant strain Gam2 with a pair of primers, Asta F1 as the forward and Asta R4 as the reverse primer. Specific primers used for astaxanthin synthetase gene amplification are listed in Table 1.

**Table 1:** The specific primers used for *crtS* gene amplification.

Primer	Sequence	Size (bp)	Position on the *crtS* gene
Asta F_1_	ATG TTC ATC TTG GTC TTG CTC	21	560-580
Asta R_1_	AGT CTG GTT GCC TTC TTT TC	20	1489-1508
Asta F_2_	GTG AAA AGA AGG CAA GA	20	1487-1506
Asta R_2_	CAT GTC AAG ACT GTC GAA GAA	21	2461-2482
Asta F_3_	CTT CGA CAG TCT TGA CAT GG	20	2463-2482
Asta R_3_	AAA GAA GCA CAG AAG AGG AAG	21	2923-2943
Asta F_4_	CGG CTT CCT CTT CTG TGC	19	2920-2938
Asta R_4_	CAT TCG ACC GGC TTG ACC T	19	3707-3725

The primers were designed according to the published sequences of astaxanthin synthetase gene. (20) with the Gen Bank accession number DQ002006. The corresponding gene fragment was amplified in the PCR reaction. Amplifications were performed in 20 µl reaction mixtures containing: 1x PCR buffer (0.1% Triton X-100, 50 mM KCl, 10 mM Tris- HCl pH 9.0 and 0.5 mM MgCl_2_), 100 ng of genomic DNA, 160 µM of dNTPs, 0.16 µM of forward primer, 0.16 µM of reverse primer, 1.6 mM of MgCl_2_ and 0.4U of Taq DNA polymerase (Cinnagen, Tehran, Iran). PCR was carried out in a PTC-1148 programmable thermal controller (BioRAD, USA). The reaction for the amplification of astaxanthin synthetase gene fragment1 in mutant strain Gam1 was run for 35 cycles with the following conditions: An initial denaturation at 94°C for 4 min, denaturation at 94 °C for 30 s, annealing at 54 °C for 30 s, and extension at 72 °C for 2 min and a final extension at 72 °C for 10 min.

PCR program for the amplification of astaxanthin synthetase gene fragment2 in the mutant strain Gam1 was set the same as the PCR program used for fragment1. Only the extension was set at 72 °C for 1 min and 30 s.

PCR program for astaxanthin synthetase gene amplification in the mutant strain Gam2 was run for 35 cycles with the following conditions: an initial denaturation at 94°C for 4 min, denaturation at 94 °C for 30 s, annealing at 54 °C for 30 s, and extension at 72 °C for 3 min and 30 s and a final extension at 72 °C for 10. For sequencing, the amplified products were electrophoresed on a 0.7% (w/v) agarose gel in TBE buffer (0.89 M Tris-base pH 8.2, 0.89 M Boric acid, and 0.02 M EDTA). Gels were stained by ethidium bromide (0.5 µg/ml), visualized and photographed in a gel doc system (UVP, UK). After detection, the desired products were extracted and purified using a DNA Extraction Kit (Cinnagen, Tehran, Iran).

**Fig. 1:**
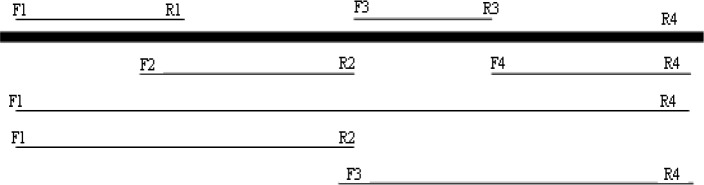
The position of the pairs of primers on the *crtS* gene, used for PCR amplification and sequencing.

Finally, the obtained DNA fragments were sequenced. The amplified fragments were sequenced using primers Asta F_1_, Asta R_1_, Asta F_2_, Asta R_2_, Asta F_3_, Asta R_3_ and Asta F_4_, Asta R_4_. The position of each primeron the *crtS* gene has been shown in Fig. 1 and Table 1.

### Astaxanthin synthetase gene alignment

A multiple sequence alignment for the reference and the mutant strains Gam1 and Gam2 was performed using the program ClustalW (http://www.ebi.ac.uk/ClustalW), and sequence identities were calculated using Gene Doc (http://www.pse.edu/biomed/genedoc).

Nucleotide sequence data were compared against the GeneBank nucleotide sequence database (BLAST search). The nucleotide sequences were analyzed using the BLAST program (http://www.ncbi.nlm.gov/BLAST).

## RESULTS

In the previous study we obtained 10 *Phaffia rhodozyma* mutant strains using gamma rays. Two mutant strains, Gam1 and Gam2 produced 15887.50 and 8972.02 µg/L of astaxanthin compared to the 1061.64 µg/L of the parental type (16). Based on their astaxanthin overproduction, the *crtS* a key gene in astaxanthin biosynthetic pathway was chosen for further studies and characterisation.

**Fig. 2:**
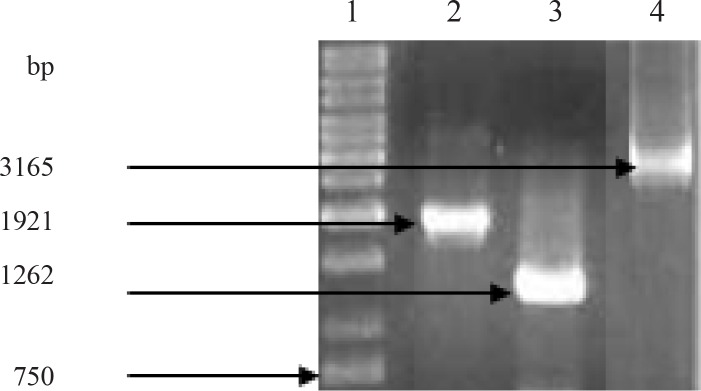
The amplification of *crtS* gene. Lane 1, 1 kb ladder, lane 2 and 3 two *crtS* gene fragments amplified in Gam1 and lane 4, the amplification of whole *crtS* gene in Gam2.

In Gam1 mutant, despite several attempts using asta F1 and asta R4 primers, the *crtS* gene was not amplified as a whole. Therefore, primers were designed and tried to amplify the gene sequence in two fragments. Using two pairs of primers, asta F1, asta R2 and asta F3, asta R4, two fragments of 1921 and 1262 bp were amplified, respectively (Fig. 2).

**Fig. 3:**
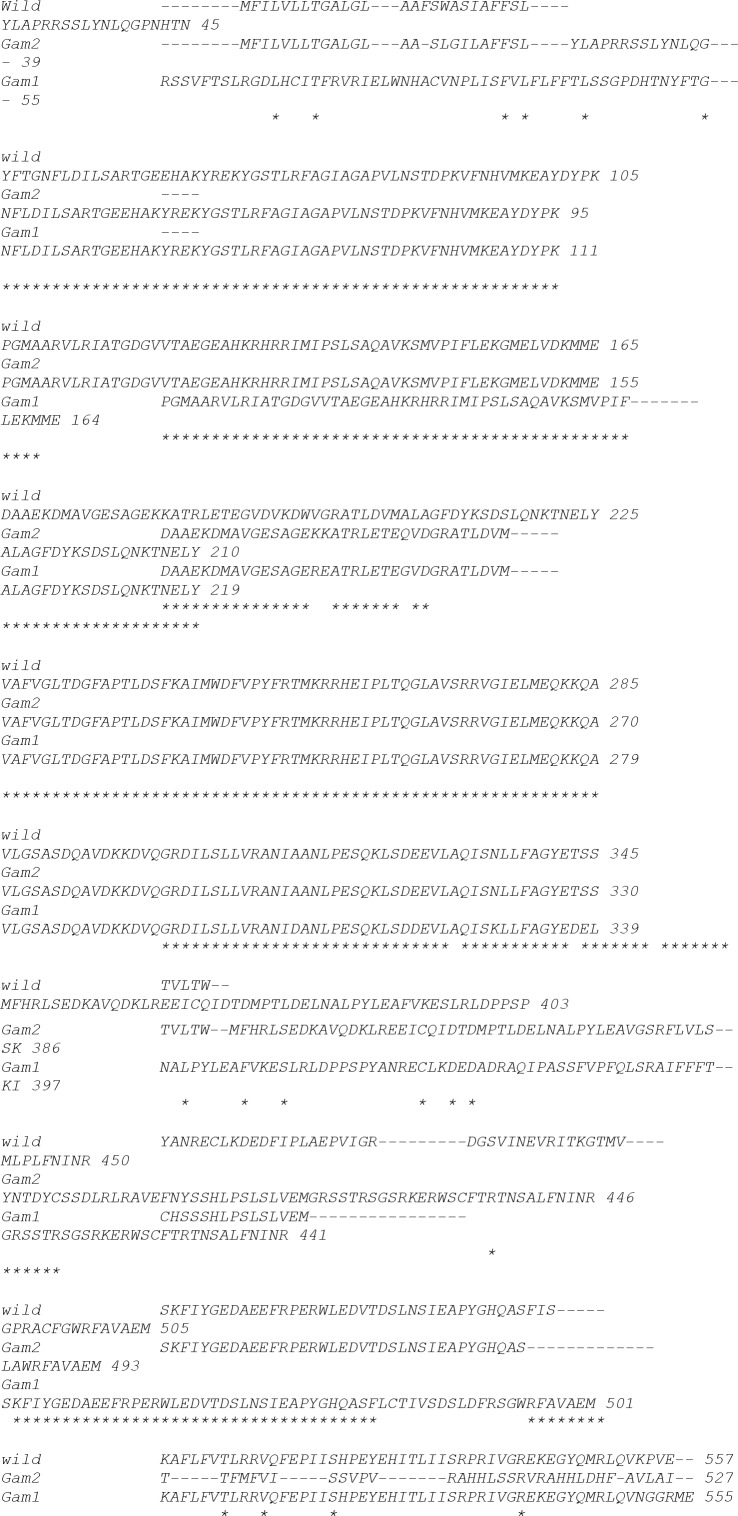
Multiple sequence alignment of astaxanthin synthase gene (*crtS*) of the reference sequence with those of Gam1 and Gam2 mutant strains using ClustalW.

Upon sequencing of both fragments, the sequences overlapping between the two fragments were omitted and the whole sequence of the *crtS* gene was deduced by joining the remaining sequences in a correct order. However, in Gam2, we were able to amplify this gene (3165 123.bp) using asta F1 and asta R4 (Fig. 2). The amplified products were aligned using the ClustalW software and mutation points were identified (Fig. 3) Then a comparison was made between reference strain with the mutant strains Gam1 and Gam2 (Fig. 3). In this comparison it was determined that homology percentage of the reference sequence with the mutant strains Gam1 and Gam2 sequences was 78% and 83%, respectively and the homology percentage of the sequences between the two mutant strains Gam1 and Gam2 was 75%.

Comparison of astaxanthin synthetase gene sequence between the reference sequence and the mutant strains Gam1 and Gam2 revealed that the reference sequence differed in 22% and 17% with the mutant strains Gam1 and Gam2, respectively. It could be said that mutations occurred in almost 20% of astaxanthin synthetase gene sequence in these two mutant strains. In DNA sequence comparison, Gam1 and Gam2 strains showed 98% and 99% homology with the reference sequence and 97% homology with each other (data not shown). These two mutant strains showed a much higher astaxanthin production potential in relation to the parental type (16).

## DISCUSSION

The astaxanthin synthetase gene (*crtS*) size consists of 3995 bp and is one of the key genes in astaxanthin biosynthesis. The product of *crtS* gene is suggested to have a hydroxylase-ketolase bifunctional activity in the conversion of ß-carotene to astaxanthin in *Phaffia rhodozyma* (12). In a complementation study of *X. dendrorhous* mutants and the expression analysis in *Mucor circinelloides* conducted by Alvarez *et al*. (10), it was reported that the CrtS enzyme is a beta-carotene hydroxylase of the P-450 monooxygenase family that converts beta-carotene to the hydroxylated derivatives beta-cryptoxanthin and zeaxanthin, but it does not form astaxanthin or the ketolated intermediates in this fungus. Ojima *et al*. (13), also cloned astaxanthin synthase gene from *Xanthophyllomyces dendrorhous*. They declared that the cDNA of this beta-carotene oxygenase encodes a cytochrome P450 monooxygenase belonging to the 3A subfamily. P450-specific domains were identified including a cytochrome P450 and an oxygen binding motif. Electrons are provided by a cytochrome P450 reductase. Functional characterisation of the enzyme by genetic modification of *X. dendrorhous* demonstrated that this P450 monooxygenase is multifunctional and catalyzing all steps from beta-carotene to astaxanthin formation by the oxygenation of carbon 3 and 4.

However, this idea is challenged by Alcaino *et al*. (12), who suggested that the conversion of beta-carotene into astaxanthin requires two enzymes, astaxanthin synthase or CrtS, encoded by *crtS* gene, belonging to the cytochrome P450 protein family, and the *crtR* gene product, from *X. dendrorhous* yeast, which encodes a cytochrome P450 reductase (CPR) that provides CrtS with the necessary electrons for substrate oxygenation.

In this research, even though the *crtS* gene and the deduced proteins were shown to have been altered due to the gamma irradiation in Gam1 and Gam2 strains, but astaxanthin is being produced and even with a much higher efficiency. Considering these facts together, it is not clear for us yet that (a) these altered CrtS proteins have found a better functionality or (b) there is an alternative pathway (s) for astaxanthin biosynthesis in this yeast as proposed by An *et al*. (21), or (c) there may be other copies of *crtS* gene compensating for the altered *crtS* gene, as the genome sequence of *Phaffia rhodozyma* is not completely known, to analyze the complete transcriptome and proteome (22, 23).

Regarding the occurrence of extra copies of some genes in *Phaffia rhodozyma*, lines of evidence come from Medwid (24), whose findings suggested that *Phaffia rhodozyma* is not haploid and also from Niklitschek *et al*. (25), who cloned and studied the genes (*idi*, *crtE*, *crtYB*, *crtl* and *crtS* at their genomic and cDNA level, controlling the astaxanthin biosynthetic pathway in the wild-type ATCC 24230 strain of *Xanthophyllomyces dendrorhous*. The genetic analysis of the *crtYB*, *crtl* and *crtS* loci in the wild-type, gave evidence of the diploid constitution of ATCC 24230 strain. In order to discover the possibility of the existence of alternative pathways or the spare copies of the genes in astaxanthin biosynthesis, we propose that site directed mutagenesis be performed on each one of the genes involved in its biosynthesis and follow astaxanthin production in the downstream.
